# WALANT Proximal Row Carpectomy Under Field Sterility: A Case Report and Description of Technique

**DOI:** 10.1177/22925503241268883

**Published:** 2024-08-18

**Authors:** Brett Ponich, Alexander Platt, Madison Turk, Maleka Ramji, Aaron Knox

**Affiliations:** 1 Division of Plastic and Reconstructive Surgery, 2129 University of Calgary, Calgary, Alberta, Canada; 2 Cummings School of Medicine, 2129 University of Calgary, Calgary, Alberta, Canada; 3 2129 Department of Surgery, University of Calgary, Calgary, Alberta, Canada

**Keywords:** WALANT, field sterility, proximal row carpectomy, WALANT, champ stérile, carpectomie proximale

## Abstract

Field sterility and Wide Awake Local Anesthesia No Tourniquet (WALANT) technique is a commonly used operative technique for many hand and wrist pathologies. We present a case of a successful proximal row carpectomy using WALANT and field sterility in a minor surgery operating theatre setting. These techniques can be applied in appropriate patients to reduce the overall cost of surgery, anesthetic complications, and prolonged hospital stay.

## Résumé

Le champ stérile et l’anesthésie locale sans garrot (WALANT, d’après l’acronyme anglais) sont une technique opératoire courante pour de nombreuses pathologies de la main et du poignet. Les chercheurs présentent le cas d’une carpectomie proximale réussie à l’aide de la technique de WALANT et du champ stérile dans un bloc opératoire pour chirurgie mineure. Ces techniques peuvent être utilisées auprès de patients bien choisis pour réduire le coût global de l’opération et le risque de complications anesthésiques et pour éviter un séjour hospitalier prolongé.

## Background

Osteoarthritis (OA) of the wrist can be painful, debilitating, and affects 1%–2% of the general population.^1^ This condition is caused by various etiologies, including scapholunate advanced collapse (SLAC), scaphoid non-union advanced collapse (SNAC), or Kienbock's disease.^2^ Surgical intervention is often necessary for treatment, and proximal row carpectomy (PRC) has been reported to improve pre-operative pain, while preserving functional mobility and grip strength.^3–5^ Furthermore, PRC is a favored procedure in older patients^6^ who often have comorbidities that can contribute to increased anesthetic risks. Wide Awake Local Anesthesia No Tourniquet (WALANT) surgery is an established method for treating hand pathologies. Studies have demonstrated the benefits of this technique through shorter procedure times, decreased recovery period, and reduced health care spending, with no associated increased risk to post-operative infection rates.^7–10^ In this case report, we highlight our reproducible technique for PRC performed with field sterility using WALANT technique.

## Methods

Electronic medical records were used to gather information regarding history and imaging. Verbal consent was obtained for use of the patient's imaging for public presentation and documentation.

**Figure 1.**
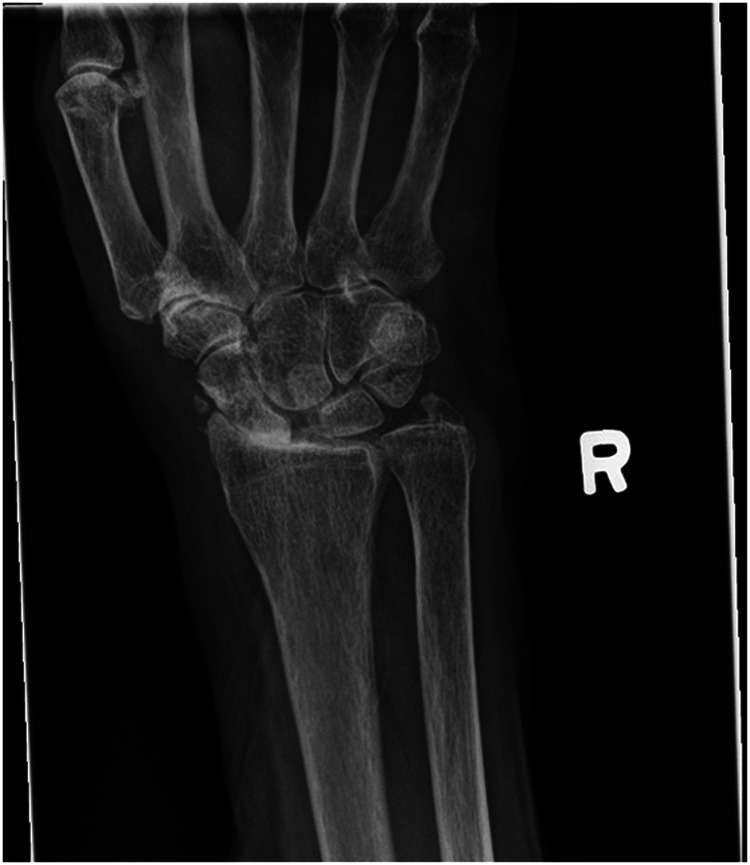
Preoperative right hand plain film posterior to anterior view x-ray.

**Figure 2.**
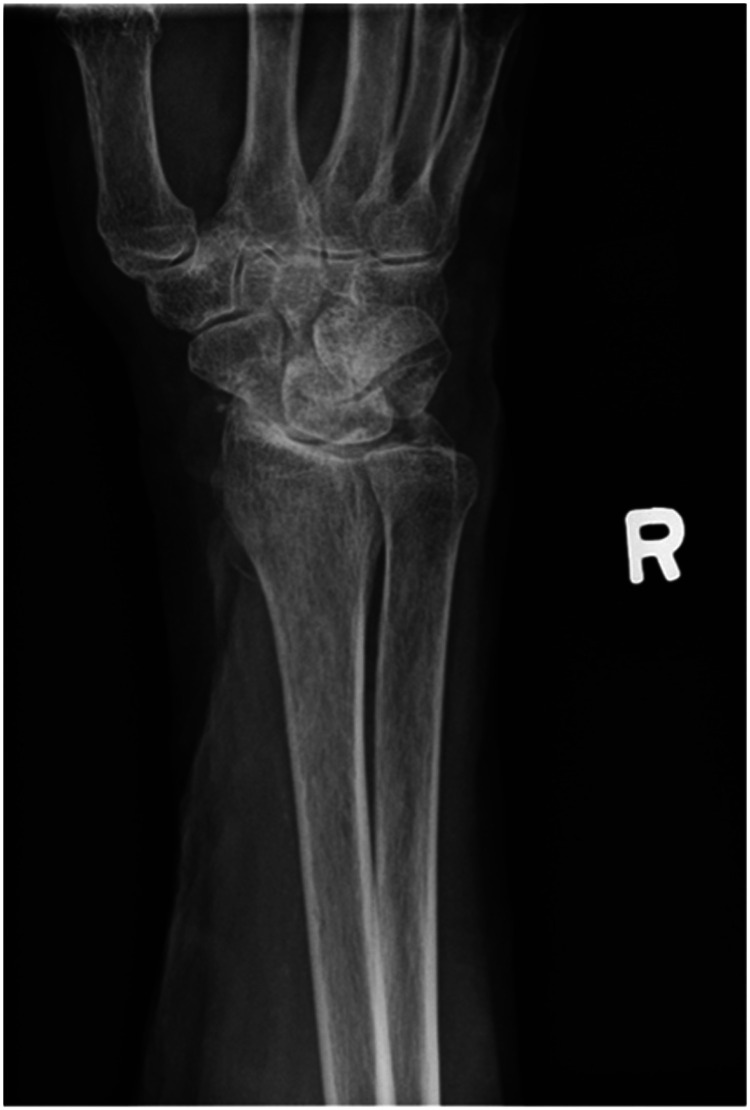
Preoperative right hand plain film oblique view x-ray.

**Figure 3.**
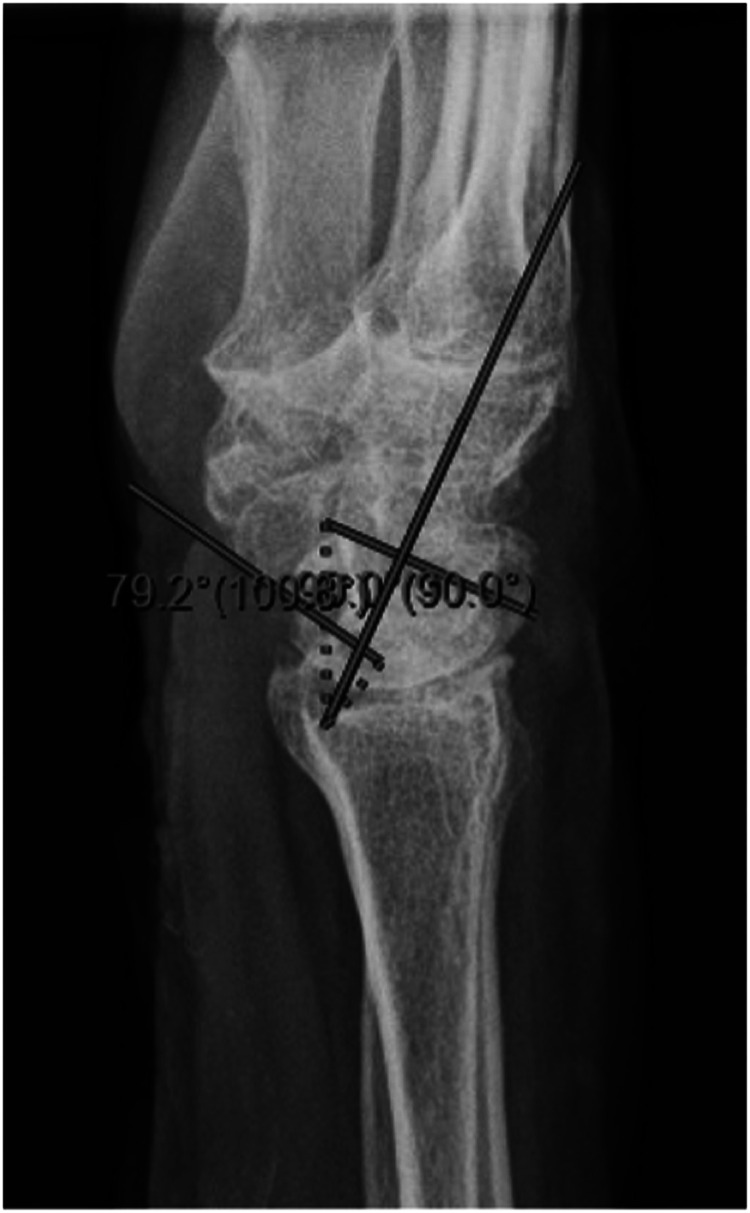
Preoperative right hand plain film lateral view x-ray.

We report a case of a comorbid 83-year-old female presenting with SLAC wrist. The patient reported new onset right wrist pain following moving a mattress two months prior. Upon presentation, radiographs demonstrated SLAC stage III arthritis with advanced radio-scaphoid OA and degenerative change between capitate and lunate (Figures 1–3).

The patient had previously trialed conservative management including splinting, activity modification, oral pain medication, and radio-scaphoid intraarticular injection of cortisone with no effect.

Upon consultation with the patient, it became clear that she would likely benefit from proximal row carpectomy (PRC) and wrist denervation for management of her symptoms. Due to her frailty and comorbidities, along with two previous surgeries under general anesthetic that had resulted in post-operative delirium and prolonged length of hospital stay, a general anesthetic OR was of concern. Therefore, surgery was planned to be done under WALANT technique with field sterility in attempt to avoid post-anesthetic complications.

## Operative Technique

The patient was not required to fast the day of surgery. She was placed onto the operating table in the supine position in the minor surgery operating theatre.

### Nerve Blocks

Local anesthesia was achieved using approximately 20cc of 1% xylocaine with epinephrine in circumferential field block fashion in addition to targeted ulnar, median nerve, anterior and posterior interosseous nerve blocks as previously described.^11^

### Equipment & Draping

The patient was then left for approximately 15 min for the anesthetic to take effect. This time was used for prepping and draping, as well as preparing necessary equipment.

The patient was prepped from proximal forearm to fingertips with chlorhexidine and wrapped with two sterile towels. A sterile bowel bag was placed on the mini fluoroscopy image intensifier and a minor surgery plastic tray was opened. The surgeon wore generic nonsterile surgical scrubs, a mask, and sterile gloves.

### Exposure and Wrist Denervation

A 10 cm longitudinal incision centered over Lister's tubercule was incised. Blunt dissection was performed to the extensor retinaculum with wide subcutaneous undermining radially and ulnarly for wrist denervation. The fourth compartment was incised and a posterior interosseus nerve (PIN) neurectomy was performed.

Dissection was continued proximally along the interosseous membrane before making a longitudinal incision and dissecting through to find the anterior interosseous nerve (AIN) on the deep surface of the pronator quadratus. Similarly, an AIN neurectomy was performed completing the wrist denervation.

### Carpal Bone Excision

The dorsal wrist approach was utilized as previously described.^12^ The lunate fossa and the proximal aspect of the capitate were assessed and noted to be free arthritic changes. The triquetrum, lunate, and scaphoid were then removed using a combination of sharp excision, rongeur, and monopolar cautery.

The capitate was then positioned to sit into the lunate fossa. The patient was taken through AROM and PROM. With radial translation there was some contact of the trapezium on the radial styloid and thus a radial styloidectomy was performed with an osteotome and a mallet. Care was taken to preserve the volar radioscaphocapitate ligament.

Fluoroscopy was then used to confirm proper position and alignment of the remaining carpus. Hemostasis was achieved with monopolar and bipolar cautery. The capsular flap was re-inset and the extensor tendons were then replaced into their native position with the exception of extensor pollicus longus, which was left out of its compartment. The extensor retinaculum was reapproximated. Verification was made that wrist and fingers could move freely before again irrigating and achieving final hemostasis. Skin closure was performed in a multilayer fashion.

The patient was placed in a dressing and a volar wrist splint in the position of safety.

Overall, the patient tolerated the procedure well and there were no complications. Total time of procedure, with local anesthetic administration to patient departure, took approximately 60 min. They were discharged home with instructions to follow up in 3 weeks time. They were referred to hand therapy for finger range of motion, edema management, desensitization, and scar massage.

## Results

**Figure 4.**
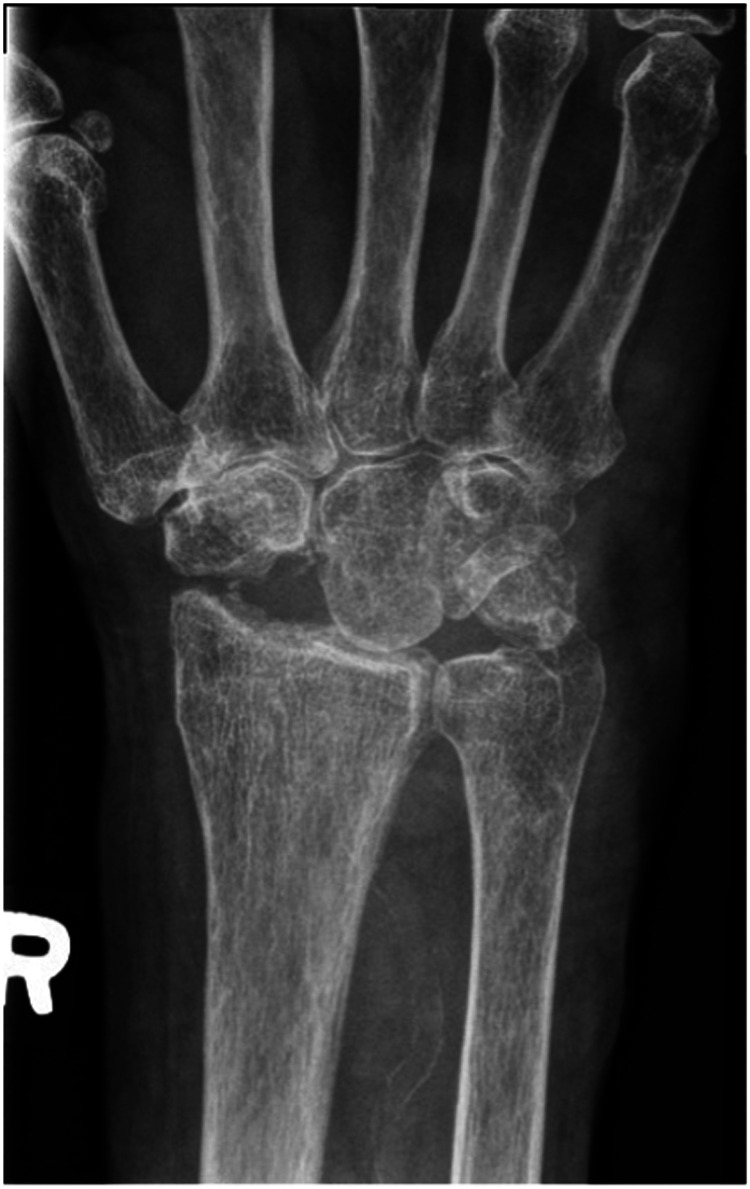
Postoperative right hand plain film posterior to anterior view x-ray.

**Figure 5.**
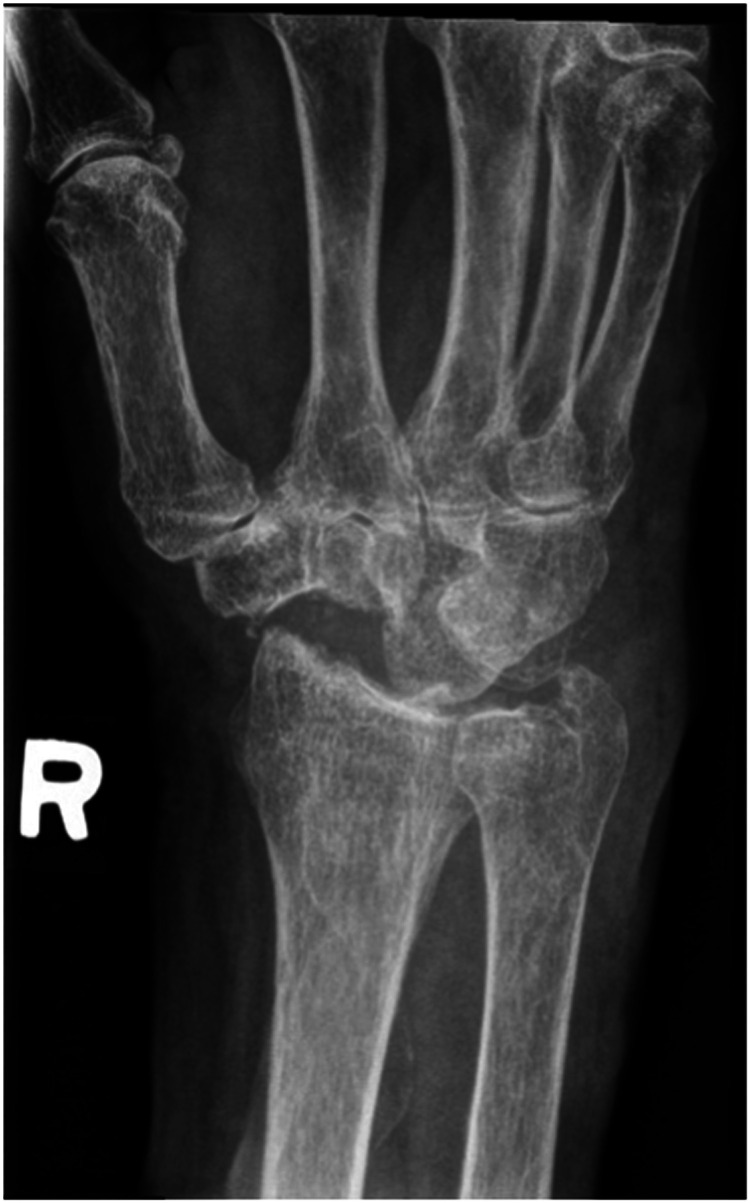
Postoperative right hand plain film oblique view x-ray.

**Figure 6.**
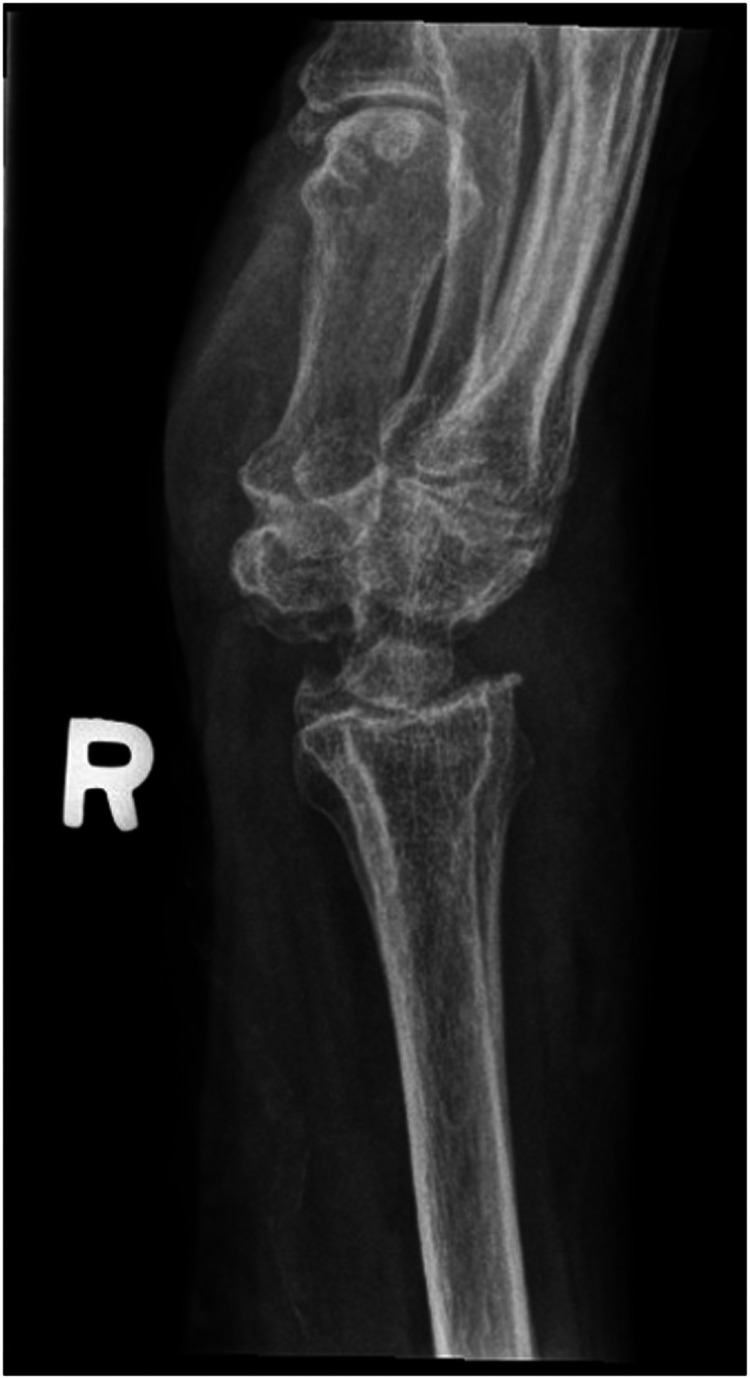
Postoperative right hand plain film lateral view x-ray.

**Figure 7.**
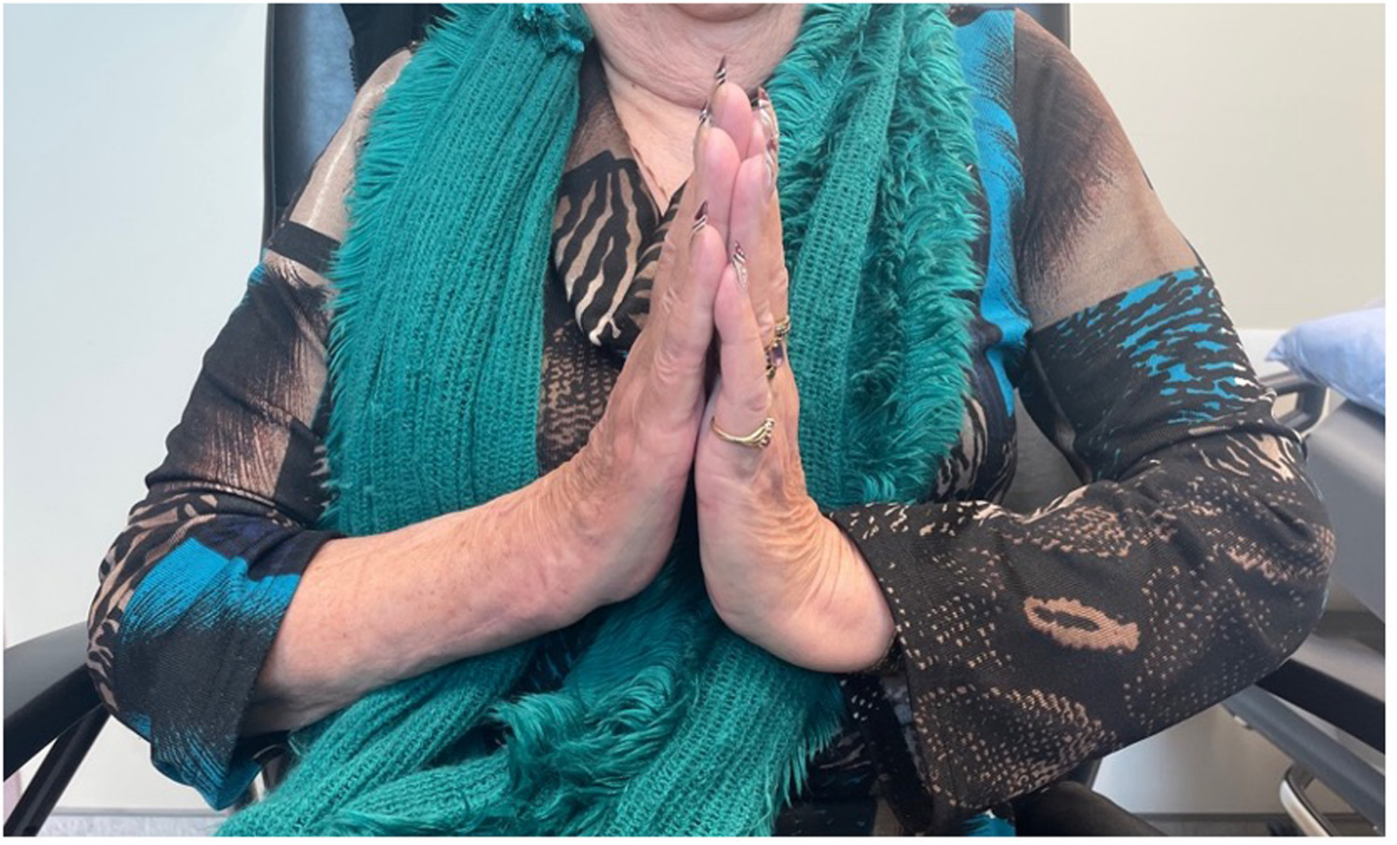
Postoperative view of wrist extension.

The patient was seen in follow up at 3 weeks with minimal pain, mild wrist stiffness and good finger range of motion. There was no evidence of infection. She was seen again for imaging and assessment at 8 weeks (Figure 4–6), and again at 4 months post-operatively. At both appointments she was pleased with her improving range of motion and increasing strength (Figure 7) (Table 1).

**Table 1.** ROM at 8 Weeks Postoperatively

Wrist ROM	AROM	
	Right	Left
Extension	41	72
Flexion	28	44
Radial Deviation	16	22
Ulnar Deviation	20	42

*In degrees.

## Discussion

WALANT surgery technique under field sterility continues to be a viable option for performing hand and wrist surgery. This is the second case we have presented that allows efficient use of hospital resources.^2^ In addition to resource conservation, this patient was able to undergo PRC and the treatment of her symptoms while being spared the increased general anesthetic risks that accompany her frailty and comorbidities.

A successful PRC was performed under WALANT and field sterility technique in a patient who had previously demonstrated increased susceptibility to general anesthetic complications. WALANT and field sterility technique continues to be a safe option while simultaneously allowing a reduction in sterile materials, efficiency in time management, and avoiding admission to hospital.
